# Exploring the role of gender and women in the political economy of health in armed conflict: a narrative review

**DOI:** 10.1186/s12992-021-00738-9

**Published:** 2021-08-04

**Authors:** Kristen Meagher, Bothaina Attal, Preeti Patel

**Affiliations:** 1 Research for Health Systems Strengthening in northern Syria, Conflict and Health Research Group (CHRG), Department of War Studies, King’s College London, London, UK; 2 Faculty of Medicine and Health Sciences, Sana’a University, Yemen and Centre for Business Research, University of Cambridge, Cambridge, UK

**Keywords:** Gender, Women, Political economy, Health, Health systems, Conflict, Peace

## Abstract

**Background:**

The ripple effects of protracted armed conflicts include: significant gender-specific barriers to accessing essential services such as health, education, water and sanitation and broader macroeconomic challenges such as increased poverty rates, higher debt burdens, and deteriorating employment prospects. These factors influence the wider social and political determinants of health for women and a gendered analysis of the political economy of health in conflict may support strengthening health systems during conflict. This will in turn lead to equality and equity across not only health, but broader sectors and systems, that contribute to sustainable peace building.

**Methods:**

The methodology employed is a multidisciplinary narrative review of the published and grey literature on women and gender in the political economy of health in conflict.

**Results:**

The existing literature that contributes to the emerging area on the political economy of health in conflict has overlooked gender and specifically the role of women as a critical component. Gender analysis is incorporated into existing post-conflict health systems research, but this does not extend to countries actively affected by armed conflict and humanitarian crises. The analysis also tends to ignore the socially constructed patriarchal systems, power relations and gender norms that often lead to vastly different health system needs, experiences and health outcomes.

**Conclusions:**

Detailed case studies on the gendered political economy of health in countries impacted by complex protracted conflict will support efforts to improve health equity and understanding of gender relations that support health systems strengthening.

## Key Messages

- Gender is a fundamental yet hugely neglected component in the political economy of health in armed conflict.
- There is limited evidence on the role of gender in post-conflict health systems but not in active conflict and humanitarian crises.
- Employing a gendered lens to political economy of health analysis will advance gender equity and equality in conflict settings.
- Women’s inclusion in the political economy of health in conflict has greater dividends for sustainable peace and more equitable social economic recovery in the post-conflict period.
- Women’s meaningful participation in peace processes creates more legitimate representation of socio-economic and health concerns.

## Introduction

The larger the gender gap, defined as the differences in experiences and opportunities between men and women, the more likely a country is to be involved in violent conflict [1, 2]. Furthermore, higher levels of gender equality, sometimes measured by fertility rates and female-to-male primary school enrolment ratios, increases the likelihood of peaceful means of addressing population-level grievances [3, 4]. The ripple effects of protracted armed conflicts and humanitarian crises such as in Yemen, the Sahel region, Ethiopia, Afghanistan, Syria and elsewhere include: extreme poverty, higher debt burdens, food insecurity, increasing number of female-headed households, deteriorating employment prospects, especially for women, increasing informal labour, and significant gender-specific barriers to accessing essential services such as health, education, water and sanitation [5]. It is estimated that 60 % of preventable maternal deaths take place in settings of conflict and displacement; women and children make up around 80 % of internally displaced populations; displaced women face unique challenges in accessing healthcare, which is linked to rising mortality rates from maternal complications of unplanned pregnancies and unattended childbirth in displacement settlements [6].

The main aim of this study is to consider and develop an understanding of the role of gender, specifically women, in the political economy of health in armed conflict settings. This article combines existing literature from a range of academic disciplines on three main subject areas: gendered and feminist political economy, gender and peace including peacebuilding, and health systems research in active conflict and post-conflict settings. It aims to enhance understanding of the importance of employing gender analysis within the wider literature on the gendered political economy, and furthermore strengthen the nascent literature of the political economy of health in conflict by ensuring it is not gender blind and envisions gender as a core component in its analysis [7]. In contextualising the gendered political economy of health in conflict settings, we seek to understand the impact of gender norms and what the value is of incorporating gender in the political economy analysis of health in conflict. For the purposes of this review, we define armed conflict as a contested incompatibility that concerns a government and/or a territory where the use of armed force between two or more parties, of which at least one is the government of a state, results in at least 25 battle-related deaths in one calendar year [8]. Given the limited literature base concerning the political economy of health in conflict, we also include post-conflict health literature. Post-conflict is defined as a conflict situation in which open warfare has ended, sometimes through a peace agreement and/or ceasefire. These situations remain tense for years or decades and can easily relapse into large-scale violence [9].

From a health systems perspective, the political economy of health in conflict examines the historical development and policies of health systems, the role and impact of the multiplicity of actors, including government, non-state actors, the private sector, international donors and humanitarian agencies, and their subsequent effects on socioeconomic health policies and health outcomes [10]. Analysing the political economy of health systems and policy can therefore contribute to improving understanding of the broader forces that affect the distribution of health-related resources within and across populations [11]. To fully comprehend the nuanced interactions between these components of the political economy, gender is fundamental. However, it is absent from the literature on the political economy of health in armed conflict and has been described as invisible from political economy more widely [12]. Gender is a social construct that refers to the characteristics of men and women, including associated norms, behaviours, expectations and roles. While the concept of gender in health varies in different societies and over time, it is a power relation that plays a critical role in determining the structural location and subjective experience of women and men in health. Furthermore, gender interacts with but is different from sex, which refers to the biological attributes of men, women and non-binary or intersex people [13]. Gender norms are described as the often unspoken rules that govern the attributes and behaviours that are valued and considered acceptable for men, women, and gender minorities. Norms are embedded in institutions, defining who occupies leadership positions, whose contributions are valued, and whose needs are accommodated [14]. Building on this, we define gendered political economy as analysis explicitly examining how society is inherently structured by gender and the subsequent inequalities that shape people’s access to power and resources, further ensuring that the perspectives of women inform the process, content and use of the analysis [12, 15]. Taking into account these definitions, and for the purposes of this study, we employ the term *gendered political economy of health in conflict.* We define this concept as the *gendered analysis of the causes of disease distribution and relative access to health services requiring attention to the political and economic structures, processes and power relations that produce socially constructed patterns, norms and structures (of health, disease, and wellbeing) to explain women’s largely subordinate role within these structures *[12, 16]. This definition is informed by feminist scholarship, which is concerned with gender inequalities that arise from a system of patriarchy and interprets society as gendered in such a way that women and men have fundamentally different experiences and access to power and privilege [17]. It should also be noted that the term feminist political economy is widely utilised, sometimes interchangeably with gendered political economy. Feminist political economy defines the material and cultural discrimination against girls and women as the primary factors that influence their social conditions and health [17]. In acknowledging that women do not form a homogenous group and that their lived experiences differ, our definition is further informed by intersectional analysis [18]. Intersectionality is defined by the idea that individuals are shaped by the interaction of different social locations such as race/ethnicity, indigeneity, gender, class, sexuality, geography, age, disability/ability, migration status, religion. These interactions occur within a context of connected systems and structures of power, for example health systems or armed conflict contexts [18]. While the application of a gendered political economy lens in health in conflict settings is complex, it enables us to develop a deeper understanding of how political, economic and social structures operate and govern the distribution of resources, benefits, privileges and authority, that reinforce gender inequity and inequality in health [19, 20]. The ultimate purpose of applying a gender lens to political economy analysis is to therefore create health systems during conflict that lead to equality and equity across not only health, but related sectors and systems, that may in turn contribute to sustainable peace building.

## Methodology

This study is a narrative review comprising both published and grey literature from a range of academic disciplines such as political science, global public health, international political economy, political philosophy and sociology, gender and feminist political economy, humanitarian, conflict and post-conflict studies. Given the limited literature base supporting this topic, a narrative review was deemed most appropriate as it provides a comprehensive overview of the concepts and wider literature contributing to this specific area, incorporating a diverse range of sources and knowledge-bases, to deepen understanding of this complex topic [21]. English and Arabic literature was obtained by searching PubMed, Scopus and Google Scholar databases. We searched literature from 2000 to 2020 as this period reflects two decades since the first global commitment to Women, Peace and Security with United Nations Security Council Declaration 1325 in 2000 to the year 2020 when we conducted the review. Specific thematic and country based sources were obtained from organisations such as the ReBUILD Consortium (a UK Government funded initiative which conducts research on stronger health systems during and after crisis); the Health Systems in Fragile and Conflict-Affected States Thematic Working Group (TWG-FCAS: https://healthsystemsglobal.org/thematic-groups/fragile-and-conflict-affected-settings/); NIHR Research Unit on Health in Situations of Fragility (https://www.qmu.ac.uk/research-and-knowledge-exchange/research-centres-institutes-and-knowledge-exchange-centres/institute-for-global-health-and-development/nihr-research-unit-on-health-in-situations-of-fragility-ruhf/); the WHO Collaborating Centre for health systems in fragile and conflict affected contexts at the Royal Tropical Institute (https://www.kit.nl/health-system-strengthening-in-fragile-states/). Relevant grey material was obtained from appropriate multilateral bodies, intergovernmental and non-governmental organisations. Examples of these sources included: World Health Organisation (WHO), other United Nations (UN) agencies (such as UN Women, UN Population Fund), the World Bank, the International Monetary Fund (IMF), the International Labour Organisation (ILO), the World Economic Forum (WEF), CARE, Oxfam, and the Gender and Development Network (GADN).

Search terms included combinations of the following: gender, women, men, feminist, masculinity, gender blind, political economy analysis, political economy of health, political economy of health in conflict, political economy of war, war economy, patriarchy, vulnerability, economic vulnerability, determinants of vulnerability, economic shocks, economic stressors, socio-economic stressors, economic recovery and conflict, humanitarian and political economy of conflict, informal economy, human rights, gender rights, health decision making, health systems strengthening, humanitarian crises, armed conflict, and protracted conflict.

The inclusion criteria for our analysis were low and middle-income countries in active armed conflict and affected by humanitarian crisis between 2000 and 2020; this also included low and middle-income countries considered as post-conflict. Definition of low and middle-income were informed by World Bank data as of 2021 [22]. Our list of conflict-affected countries (countries that were in conflict or post-conflict at some point between 2000 and 2020) included: Afghanistan, Angola, Armenia, Azerbaijan, Bangladesh, Benin, Burkina Faso, Burundi, Cameroon, Central African Republic, Chad, Colombia, Congo, Democratic Republic of Congo, Eritrea, Ethiopia, Iraq, Liberia, Libya, Mali, Myanmar, Nepal, Niger, Nigeria, Sierra Leone, Somalia, Sri Lanka, South Sudan, Sudan, Syria, Timor Leste, Uganda, Ukraine, Venezuela, and Yemen. This list of countries was obtained from the Uppsala Conflict database (https://ucdp.uu.se/year/2019) with additional information used from the Armed Conflict Location & Event Data Project (ACLED): https://acleddata.com/data-export-tool/ We also included low and middle-income regions in conflict: the Sahel, Nagorno Karabak, Palestine. These countries have remained low or middle-income during 2000–2020.

We excluded high-income countries including those that host refugees as this requires a separate level of analysis.

## Results

Interpretation and synthesis of the relevant literature was used to capture the results below. Four broad areas emerged from a mixture of theoretical and empirical research: definitions and content of a gendered political economy and why it is important in health and conflict; the widespread health and non-health effects of armed conflict on women; the importance and lessons from work on gendered health systems in conflict and fragile contexts; and lastly, the relation between gender equality and sustainable peace. These are presented in turn below.

### Applying a gendered political economy framework to conflict

Within the broader disciplines of political economy and related feminist theories, there are several theoretical frameworks which are useful for understanding what political economy of health in conflict means, how gender fits into this and why it is important in strengthening public systems in conflict and advancing policy reform that supports peacebuilding initiatives [23]. Political economy focuses primarily on two main facets, *power* and *resources* and how they are distributed and contested (either through violent or non-violent means) in different contexts: stable, conflict and post-conflict [11, 24]. A gendered political economy provides a deeper understanding of how power relations and patriarchy hinder progress on gender equality and equity. In conflict settings, it does this by interrogating how resources are allocated across different institutions, namely public services such as health care, and ultimately how these embedded structures work to disadvantage women [23, 25]. Pia Riggirozzi’s study further contributes to a gendered framework by drawing attention to the daily experience of gender-based social relations and tensions reflecting social, political, health divisions and economic stressors which prevent women from exercising their rights to equality [26]. Gendered power differences tend to shift constantly and in many conflicts, women’s new roles as economic providers seem to especially threaten masculine authority [6]. Alsaba et al., present the only conflict-specific political economy framework which helps to analyse how gendered dimensions of the Syrian conflict interact with market and state structures to reinforce gender inequalities, exposing women to particular forms of violence, increasing women’s vulnerability and forcing them into the informal labour market and the war economy [27].

### Impact of conflict on women

Conflict effects men and women differently and exacerbates gender inequalities [27]. Men and boys make up the vast majority of direct victims of armed conflict, for example forced recruitment and arbitrary detention, while women and girls become more vulnerable to the indirect impacts of war, including sexual and gender-based violence, access to health, food, education [28]. Studies have shown conflict-affected countries since 1990 have consistently higher maternal and child mortality rates than non-conflict countries and access to essential reproductive and maternal health services for poorer, less educated and rural-based families was considerably worse in conflict versus non-conflict countries [29]. Alarmingly high rates of sexual and gender-based violence with a strong association of mental health disorders, such as Post Traumatic Stress Disorder and Depression, have long been documented in several conflict-affected settings [30, 31]. Women are disproportionately affected by the negative consequences of conflict-induced forced displacement [6]. Displaced women carry an unequal burden of care because of a gendered responsibility toward caring for children and the elderly. In many conflicts, women often are the last to flee, are usually fleeing with children, and may have to undertake in transactional sex to provide food for themselves and their children [6]. Yet the feminisation of care burdens and survival remains largely invisible in many conflicts and emergencies [32]. Most recently, with the added economic, health and social stressors of Covid-19, domestic violence cases are on the rise for women in conflict settings, such as in Yemen [33]. In the Yemen conflict, the number of female-headed households has risen considerably, forcing women to take on new roles with very limited support as many men are fighting in the conflict and are unable to return to their families [33]. Yemeni women’s increased vulnerability has led to negative coping mechanisms such as early marriages and child labour. Restrictive marital practices, gender norms, and experiences of conflict were major drivers of both partner and non-partner violence in the conflict in South Sudan [31].

### Health systems in armed conflict

From a health systems perspective, political economy examines historical development and policies, the role of influential stakeholders such as the government (or in some conflict-affected areas, quasi-governments), humanitarian agencies and donors, the private sector and community-level organisations (such as the Idleb Health Directorate in northwest Syria). Incorporating gender into the political economy of health systems illustrates how females, males and peoples of other genders live, work and relate to each other at all levels of the health system. Factors such as vulnerability to illness, health seeking behaviour, access to health services, health expenditure and financing, nature of the health workforce (including capacity strengthening), data collection and management, design and use of medical products and technology, and health policies are all heavily influenced by the gendered political economy of health in conflict [34, 35]. The ReBUILD consortium provides useful analysis of gender within its post-conflict health systems analysis. Based on research in Sierra Leone, Zimbabwe, northern Uganda and Cambodia, their findings suggest women are under-represented in health management roles, they are prone to having lower paid positions within the health system, while also having additional household caring responsibilities, and their health system experiences are further influenced by intersectional factors including geography (rural vs. urban), poverty and household structure [36]. However, regulatory reforms and frameworks tend not to sufficiently address gender concerns. Armed conflicts have distinct burdens on men and women in the health workforce; studying these differences within health systems and their relation to job type, task distribution, exposures, and health outcomes in men and women is essential [37].

### Sustainable peace and gender equality

A worrying trend from the literature is that the socioeconomic and political status of women directly impacts the likelihood of conflict. As highlighted, the larger the gender gap, the more likely a country is to be involved in inter- and intrastate conflict. Melander’s study suggests that more equal societies, measured as female representation in parliament and the ratio of female-to-male higher education attainment, have lower levels of intrastate (internal) armed conflict [38]. Studies suggest that sustainable peace and economic prosperity are more likely in countries with higher levels of gender equality and political participation during all phases of conflict – pre-conflict, active conflict and post-conflict [39]. Women’s meaningful participation in peace negotiations leads to better accord content (for health and socio-economic reforms for example), higher agreement implementation rates, and durable peace [39]. Women’s participation in economic and political life is therefore vital for conflict prevention and resolution [5].

Twenty years ago, the United Nations Security Council (UNSC) passed Resolution 1325 to support women’s participation in peace negotiations, post-conflict reconstruction, and to protect women and girls from wartime sexual violence [40]. Since then several subsequent UN Resolutions, the Convention on the Elimination of All Forms of Discrimination Against Women (CEDAW), Millennium Development Goal 3 on promoting gender equality and empowering women, and Sustainable Development Goal 5 on gender equality have all strongly endorsed a gender perspective in addressing development, conflict and humanitarian challenges. The World Health Organisation envisages health as a neutral starting point in convening the myriad of actors in conflict, as they work towards shared health and economic goals [41]. However, it is important to note that conflict is often exacerbated particularly where there is inequity in health service delivery, often reflecting more broader societal inequality. Given this understated link between health in conflict settings and peace, linking the gendered political economy of health in conflict to peace agendas is crucial. It seems quite clear that if women’s participation in peace processes is more meaningful, there is more legitimate representation of both women and men’s health and socio-economic concerns, including those that impact health systems [42].

## Discussion

Our main findings suggest that although there is emerging global health interest in the broader fields of political economy, there was very little evidence devoted to gender and specifically women in the political economy of health in active conflict and humanitarian settings. The literature furthermore appears sparse with very little case study or empirical evidence to test more developed theoretical perspectives. Very few case studies examine the broader socioeconomic impacts of health in conflict; how men and women’s livelihoods are affected and how this connects to health systems, access to health services and health seeking behaviour. Nevertheless, there was considerable theoretical discussion on what constituted a gendered political economy of health and why it is important. In its application in conflict affected settings, it was notable that most of the literature concentration was on post-conflict settings rather than those embroiled in active conflict with severe on-going gender-related conflict and humanitarian consequences. Three interrelated reflective themes are analysed below, which may provide researchers and policy-makers a platform to advance this area and specifically how it could be applied in protracted conflict settings to support monitoring and implementation efforts (such as national action plans), to endorse UNSC 1325 (and related UNSC resolutions), the World Health Organisation’s Commission on the Social Determinants of Health, the Humanitarian Grand Bargain, the Women Leaders in Health and Conflict Initiative and several related gender equality markers.

Firstly, a gendered political economy perspective is vital for understanding how power relations and entrenched patriarchy hinder progress on gender equality and equity in countries and regions affected by war. It is well established that conflict has widespread collateral effects such as destruction of the health system, adverse health outcomes for women, generational effects such as poverty [43]. A gendered political economy approach may enable researchers and policy makers to enact policies, programmes and interventions beyond mere rhetoric of global gender equality initiatives. For example, in creating the evidence base required to increase funding of initiatives that support and recognise women’s active contribution to health in conflict. Gendered analysis of the political economy of health is therefore not simply about focusing on health outcomes; it must go further to understand the relationship between the biomedical approaches, the socio-economic determinants of health, the social construction of gender and its direct impacts on structural gender inequalities [44, 45].

Despite global declarations and commitments to gender equality in conflict and humanitarian crises, several barriers remain entrenched that prevent these goals from being effectively implemented. Statistics of peace accord content and implementation rates found that agreements with women signatories demonstrate better accord quality and higher implementation rates - Yakin Ertürk, a former UN Special Rapporteur on Violence against Women, argues that “although new international gender equality instruments continue to be adopted, they remain divorced from the structural inequalities that characterise the global political economy. The comeback of the security state, rise of right-wing populism, general erosion of states’ human rights commitments and the shrinking political space for civic activism, leave these instruments deskbound” [46]. Involving women in peace negotiations is crucial but the peace table, like an active conflict setting, is overwhelmingly power-driven representing an intricate web of political and economic interests, often masked under the guise of ethnic or religious rivalries. Women’s inclusion in peace processes must also take account of the diversity that women represent, through an intersectional approach, and not treat women as a homogeneous group in any society [39]. Underlying much of the policy focus is the role of the state as being the primary agent for gender equality, yet in many conflict-affected regions, the state is also the main perpetrator of violence [47]. Civil society groups and movements are therefore incredibly important for ensuring accountability, advocating for inclusive language on gender equality in peace agreements and including provisions for women’s equal access to land, credit and productive resources, health care, and education and training [48]. By reorienting the political economy analysis of health in conflict to incorporate a gendered lens, women can be envisioned as agents of change and meaningful contributors to health systems, rather than purely victims of conflict, external to systems level changes [49].

Secondly, there is significant evidence on the gendered nature and burden of conflict and the subsequent impact on health systems. Women suffer more so than men from the damage to the health infrastructure, wider economic damage and forced displacement. In many societies the division of labour tends to be highly gendered with women taking on responsibility of households and communities, a role that is increasingly divested unto women and girls especially in the times of crisis. Women’s gendered role within the household can limit their participation in formal employment which reinforces gendered norms around women’s role as secondary and dependent, not only within the household, but also within public services and markets [44]. Under-paid labour force and lower earnings for women means they generally experience lower social status, less autonomy to make health decisions, and access to health care is often crippled by out-of-pocket payments [50]. The feminised burden of care especially during times of conflict and emergencies contributes to the heightened mortality and long-term health deterioration for women and girls [51]. Maternal mortality tends to be worse in conflict-affected countries [52, 53]. Coverage of antenatal care, skilled birth, vaccination services is reduced in conflict settings [54]. Women and children are heavily dependent on a functioning health system and vulnerable to economic and societal disruption induced by conflict. Therefore, political economy analysis must incorporate gender inclusive approaches and be rooted in contextuality to understand the barriers to accessing health resources and the impact on health systems more broadly.

Lastly, the health systems and conflict literature features discussions on gender equity and equality but only in very few post-conflict settings. Limited evidence suggests that public health measures including equitable access to basic health care may contribute to peacebuilding in the aftermath of conflict [55]. Kruk’s study argues that rebuilding health services can play an essential role in promoting social cohesion in a nation’s post conflict recovery stage yet supportive empirical evidence is thin [56]. There have been some suggestions that in the Syrian conflict, the collective deprivations experienced by some of the parties central to the conflict may be addressed through the provision of essential health care, as part of the rebuilding of a fractured society and to bring communities and warring factions together [55]. However, this fails to take into account a very complex geography of conflict with different regional political factions. Therefore, incorporating gendered political economy analysis in health systems strengthening, informed by intersectional analysis, ensures that people at all levels of health care decision making reorient their notion of wellbeing to include gender equality for women in all their diversities [57]. Taking a health systems approach reinforces the value of incorporating gender as an essential component of the political economy of health in conflict; women are disadvantaged by the structures that influence health systems in conflict and are frequently excluded from decision-making in not only health, but broader systems during active conflict, they are also disproportionately impacted by armed conflict. Therefore, cultivating and harnessing the advancements of women’s meaningful leadership, that includes decision-making, at community, national and international levels, and acknowledging the significance of their contribution to health systems strengthening in conflict and humanitarian crises is paramount. This will in turn create effective and meaningful leadership models, that influence decision-making in health systems that may in turn contribute to sustainable peace building [58].

## Limitations

This study has several limitations. Firstly, while there is very little literature available on the gendered political economy of health in conflict, there is even less country-specific analysis, which would take into account the various typologies of conflict, for example what resources are available in specific countries during active conflict varies greatly. This makes determining the need for a gendered political economy of health complicated, as context specificity would enhance its applicability. Secondly, defining the gendered political economy of health in conflict is open to considerable interpretation. This is compounded by there being no single conceptual framework for political economy analysis, the lack of a working definition of the political economy of health in conflict, as it is a novel concept, and that the feminist political economy and gendered political economy are frequently used interchangeably. Thirdly, this study does not elaborate on the role of men or individuals who identify with non-binary genders within the gendered political economy of health in conflict. While crucial in understanding the nascent concept of the gendered political economy of health in conflict, it requires further investigation which would complement not only this study, but how the concept is understood across all active conflict settings. Fourthly, this study only used search terms in English and Arabic. Further literature may be available in other languages and this may also source literature beyond the regions and countries we found. Most of the relevant studies we found were from the Eastern Hemisphere with the Middle East and North Africa region being overrepresented in the literature compared with other conflict-affected regions. Subsequently, we are unable to make conclusions on all conflict-affected regions. We therefore recommend future studies address this key limitation.

## Conclusions

The gendered political economy of health in armed conflict is a convoluted term, influenced by various concepts, theories and ontologies. Yet its importance in deepening understanding of the nexus of health, gender and armed conflict is crucial. Gender-responsive analysis remains thin within the wider field of the political economy of armed conflict [46]. Health and health systems are influenced and determined by political and economic factors, which in conflict settings is precarious. The division of health resources and the implementation of policies are driven by political interests, both internal and external. The political economy of health in conflict is therefore an important concept to understand and amalgamating a gender inclusive lens within this is crucial. Understanding a specific health systems history and context is vital in understanding how to progress strengthening it. This is particularly critical during active conflict, so that robust health systems are developed to support vulnerable populations impacted by conflict and to support the post conflict phase. While some research on the political economy of health in post-conflict settings has been conducted, very little research has focused on political economy of health during active conflict. In a world wreaked by several violent protracted conflicts, it is vital to utilise political economy analysis in order to build sustainable systems that meet the needs of populations afflicted by conflict. Unpacking the barriers to gender inequity and inequality in conflict settings that utilise context specific case studies may set the agenda for developing gender inclusive structures and systems to better support the post-conflict phase and wider peace building initiatives. Furthermore, context specific understanding of the role of women may serve as a platform to acknowledge the wider gendered impacts of conflict that attribute to a lack of meaningful participation in determining health policies and priorities for individuals with intersecting identities and who identify with non-binary genders.

## Data Availability

Not applicable.
